# The role of cytonuclear interactions to plant adaptation across a *Populus* hybrid zone

**DOI:** 10.1098/rspb.2025.1239

**Published:** 2025-11-26

**Authors:** Michelle Zavala-Paez, Brianna N. Sutara, Stephen R. Keller, Jason A. Holliday, Matthew C. Fitzpatrick, Jill A. Hamilton

**Affiliations:** ^1^ Pennsylvania State University, University Park, PA, USA; ^2^ University of Vermont, Burlington, VT, USA; ^3^ Virginia Tech, Blacksburg, VA, USA; ^4^ University of Maryland Center for Environmental Science, Frostburg, MD, USA

**Keywords:** chloroplast, nuclear, co-introgression, hybridization, adaptation, physiology

## Abstract

Co-adaptation of cytoplasmic and nuclear genomes is critical to physiological function for many species. Despite this understanding, hybridization can disrupt co-adaptation, leading to a mismatch between maternally inherited cytoplasmic genomes and biparentally inherited nuclear genomes. Few studies have examined the consequences of cytonuclear interactions to physiological function across environments. Here, we quantify the degree of co-introgression between chloroplast and nuclear–chloroplast (N-cp) genes across repeated hybrid zones and its consequences to physiological function across environments. We use whole-genome resequencing and common garden experiments with clonally replicated genotypes sampled across the natural hybrid zone between *Populus trichocarpa* and *P. balsamifera*. Geographic clines were used to test for co-introgression of the chloroplast genome with N-cp and non-interacting nuclear genes. Co-introgression of the chloroplast alongside N-cp genes was limited, although contact-zone specific patterns point to the importance of regional differences. Combining ancestry estimates with phenotypic data across common gardens revealed that mismatches between chloroplast and nuclear ancestry can influence physiological performance, but the strength and direction of these effects vary depending on the environment. Overall, this study highlights the importance of cytonuclear interactions to adaptation, and the context-dependent role the environment may play influencing that interaction.

## 1. Introduction

Organelle and nuclear genomes function together to maintain key developmental and physiological processes critical to adaptation [1–4]. In plants, these interactions are critical, as photosynthesis and respiration require coordination of nuclear-encoded proteins alongside the chloroplast and mitochondrial genomes [5–7]. Cytonuclear coadaptation predicts that nuclear genes involved in cytonuclear interactions will co-evolve with cytoplasmic genes to optimize plant function and fitness [8–10]. This promotes positive intergenomic epistasis and is considered a major force shaping cytonuclear interactions [2,11]. Recombination between sister species via hybridization can disrupt co-adaptation, leading to a mismatch in ancestry with impacts to plant fitness [8,12–15]. Moreover, environmental gradients may favour specific combinations of cytoplasmic genes and their interacting nuclear genes that optimize physiological performance under local conditions [16–18]. Thus, understanding how mutation and recombination impact cytonuclear interactions across environments will be critical to ensuring optimized plant function in response to environmental change.

Despite the importance of co-adaptation between cytoplasmic and nuclear genomes to plant function, uniparental inheritance of the cytoplasmic genome with biparental inheritance of the nuclear genome has frequently led to a mismatch in genomic ancestry where sister species hybridize [2,15,19]. As a result, hybrids may have a cytoplasmic genome from one species while exhibiting a mixed nuclear background resulting in negative intergenomic epistasis for co-adapted genes [2,15,19,20]. Indeed, where previously isolated lineages have come into secondary contact, accumulated differences have contributed to genetic incompatibilities with negative impacts on fitness [21,22]. Experimental studies have shown that such mismatches can impair metabolic processes, reduce photosynthetic capacity, contribute to chlorosis and ultimately decrease fitness [8,12–15,23]. Given these potential impacts, understanding how and when hybridization influences cytonuclear interactions and their genetic and phenotypic consequences across environments will be critical for predicting plant performance.

Despite the potential fitness consequences of cytonuclear mismatch for plants, cytoplasmic genomes frequently introgress across species boundaries and this introgression may play a role in adaptation to new environments [24–26]. Cytoplasmic introgression has been shown to enhance organellar function [15,27] and can provide a mechanism to mitigate the potential fitness consequences of high cytoplasmic mutational load [24,28,29]. However, because nuclear and cytoplasmic genomes interact, the benefits may depend on co-introgression of cytoplasmic genomes alongside their nuclear-encoded interacting genes [24,30]. Theory predicts that such genes that interact across the cytoplasm and nuclear genome should be more likely to introgress alongside cytoplasmic genomes, irrespective of nuclear genes without such interactions (hereafter, non-interacting nuclear genes) [13,24,31,32]. Since cytonuclear interactions can be shaped by local environmental conditions [16,17], evaluating patterns of co-introgression across multiple contact zones in a hybrid zone offers a unique opportunity to assess how environment influences these interactions. Despite this understanding, it remains unclear whether cytoplasmic genomes and their interacting nuclear genes co-introgress more frequently than non-interacting nuclear genes across repeated zones of natural hybridization.

*Populus* is an ideal system for studying cytonuclear interactions as repeated hybridization between sister species has led to frequent evidence of cytoplasmic introgression, resulting in natural variation in cytonuclear combinations [26,33–35]. Here, we focus on the widespread natural hybrid zone between *P. trichocarpa* and *P. balsamifera*, which spans a steep maritime-continental climatic gradient characterized by extensive nuclear gene flow and the formation of advanced-generation hybrids [36–38]. Within this hybrid zone, species-specific chloroplast haplotypes are divergent, but substantial interspecific gene flow has resulted in a spectrum of individuals with varying degrees of nuclear and chloroplast ancestries [33,36,39]. This variation provides a unique opportunity to evaluate co-adaptation between the nuclear and chloroplast genomes, specifically by testing whether nuclear-encoded genes that directly interact with the chloroplast-encoded proteins (hereafter, N-cp genes) and chloroplast genome co-introgress and by quantifying how varying cytonuclear combinations affect traits important to plant physiological function. Despite recognition of the evolutionary significance of hybridization in *Populus* and the extensive use of *Populus* hybrids in restoration and bioenergy [40,41], the role of cytonuclear interactions to adaptation remains largely untested.

Using whole-genome resequencing data from *Populus* genotypes sampled across repeated natural hybrid zones between *P. trichocarpa* x *P. balsamifera*, we test for patterns of co-introgression between N-cp genes and the chloroplast genome and evaluate the phenotypic effects of cytonuclear interactions to physiological trait variation across environments. Given its compact size, conserved gene content, and central role in photosynthesis, we use the chloroplast genome as our focal cytoplasmic genome to evaluate patterns of co-introgression, focusing specifically on nuclear–chloroplast interactions [26]. We address three main questions. (i) Is there evidence of chloroplast introgression across multiple contact zones within the hybrid zone between *P. trichocarpa* and *P. balsamifera*, and what influence does environment have on the extent or direction of chloroplast introgression? Given previous reports of extensive gene flow between *Populus* species, we predict that introgression of the chloroplast genome may be environmentally dependent. (ii) Do geographic cline comparisons between N-cp genes and the chloroplast alongside non-interacting nuclear genes indicate co-introgression for genes critical to adaptation? If cytonuclear coadaptation is maintained by selection, then N-cp genes should exhibit clinal variation that parallels chloroplast ancestry indicative of co-introgression. In contrast, non-interacting nuclear genes are expected to vary independently, exhibiting clinal patterns independent of the chloroplast genome. (iii) Do varying cytonuclear combinations influence physiological traits critical for adaptation, and if so, do these effects vary across environments? We expect that cytonuclear discordance will reduce physiological performance relative to matched combinations due to disruption of co-adapted gene complexes. By addressing these questions, our study provides new insights into how cytonuclear interactions shape adaptation, emphasizing the role of environmental selection in structuring patterns of co-introgression and trait variation in *Populus*.

## 2. Methods

### (a) Plant material sampling, genomic library preparation and variant calling

In January 2020, dormant vegetative cuttings were sampled from 574 individual *Populus* genotypes across six latitudinally distributed contact zones spanning the natural hybrid zone between *Populus trichocarpa* and *P. balsamifera* (electronic supplementary material, figures S1, tables S1–S2). Cuttings were transported to Blacksburg, VA, USA, where they were divided and propagated, creating clonally replicated rooted *Populus* cuttings. Clones were maintained in the greenhouse at 24°C daytime and 15.5°C nighttime temperatures without supplemental lighting (full details described in [36]). Following the vegetative flush, 100 mg of fresh leaf tissue was collected from rooted cuttings for DNA extraction and whole genome resequencing. Methods regarding DNA extraction, genomic library preparation and subsequent quality control and filtering associated with whole-genome resequencing are available in Bolte *et al*. [36] and resulted in 4 497 721 biallelic SNPs (electronic supplementary material, Methods).

### (b) Chloroplast genome assembly, chlorotype identification and divergence

Chloroplast genomes were assembled using NOVOPlasty [42] and annotated in Geneious v. 7.1.4 [43] using *P. trichocarpa* (NC_009143.1) as a reference (see electronic supplementary material, Methods). Of the 574 samples, 573 chloroplast genomes were successfully assembled, with one sample (GPR−14_S50_L001) failing due to low coverage (<10×), and 10 individuals removed due to assembly errors in the small single-copy region (SSC).

To infer genotypes and chloroplast ancestry, we used 563 chloroplast genomes from our collection alongside 58 *Populus* spp. and *Salix interior* and *S. tetrasperma* chloroplast genomes from GenBank (electronic supplementary material, table S3). Chloroplast genomes were aligned using MAFFT v. 7.453 [44], and a maximum likelihood (ML) tree was constructed in IQ-tree 1.6.11. The best-fit evolutionary model (TVM+F+R2) was selected using ModelFinder [45] based on the lowest BIC score, and branch support was assessed with 1000 bootstrap replicates. Ancestry was assigned based on chlorotype-specific clades using FigTree v. 1.4.13 (electronic supplementary material, figures S2 and S3). Phylogenetic assignments were validated using BLASTn searches of *mat*K and *rbc*L genes (≥99% identity; electronic supplementary material, Methods).

To assess chlorotype-specific differences between *P. trichocarpa* and *P. balsamifera*, chloroplast genomes were aligned to the *P. trichocarpa* reference chloroplast genome using MAFFT v. 7.453 [44] and SNPs with fixed allele frequency differences were identified using a custom R script. Variants were mapped to the reference genome and subsequently examined in Geneious [43] to identify synonymous and nonsynonymous substitutions.

To infer ancestral and derived alleles for fixed amino acid differences observed between chlorotypes, we aligned the corresponding three-nucleotide codons for *Populus balsamifera* and *P. trichocarpa* with previously used outgroups (*Salix* and *Populus* species). Sequences were used to polarize substitutions and determine the directionality of allele change. Codon alignments were performed in Geneious v. 2025.2.1 [43], and gene trees were constructed using the neighbour-joining method with Jukes–Cantor distances.

### (c) Testing for the role of environment on chloroplast ancestry

Logistic regression was used to assess the relationship between environment and chloroplast ancestry across the *Populus* contact zones. We used climate averages associated with genotype origin (1961−1990) for six climatic variables previously associated with nuclear genetic structure [36]. These variables include continentality (TD), mean annual temperature (MAT), mean annual precipitation (MAP), climate moisture deficit (CMD), relative humidity (RH) and precipitation as snow (PAS) downloaded from climateNA V742 [46]. Firth’s bias-reduced logistic regression was used to account for small sample sizes and to address complete separation, which occurs when predictors perfectly separate outcomes [47,48]. Logistic regression models were fitted using the logistf function in R v. 4.3.1 to quantify the strength and direction of associations between chloroplast ancestry and environmental gradients [49].

### (d) Geographic clines to compare patterns of introgression between chloroplast- and nuclear-interacting genes

Geographic clines were compared between the chloroplast genome, nuclear–chloroplast (N-cp) genes and non-interacting nuclear genes to test expectations for co-introgression across latitudinally distributed contact zones. We selected 282 N-cp genes that directly interact with chloroplast-encoded proteins previously identified in *Arabidopsis thaliana* from the Cytonuclear Molecular Interactions Reference (CyMIRA) database [50] and identified their *Populus* orthologs using BLASTP (*e*-value < 10⁻⁵, minimum identity = 60%). Single-copy nuclear genes were selected to serve as a control (hereafter non-interacting nuclear genes). These genes were selected from 2668 single-copy nuclear genes in *P. trichocarpa* after excluding all nuclear genes with known cytonuclear interactions listed in CyMIRA, resulting in 2611 nuclear genes for use in downstream analysis.

Locus-specific ancestry for each of the N-cp and non-interacting nuclear genes was inferred using Loter [51], which assigns species-specific ancestry using phased reference panels of unadmixed parental species, estimating ancestry for linkage blocks within admixed individuals. Parental-type genotypes were identified previously from [52] based on admixture with *Q* > 0.98 and *Q* < 0.02 (for *K* = 2) assigned as *P. trichocarpa* (*n* = 131) and *P. balsamifera* (*n* = 90), respectively. Local ancestry was assessed for 323 admixed individuals, and gene-specific ancestry values were extracted from the ancestry matrix using gene positions from the *P. trichocarpa* v. 4.1 reference genome, including a ±100 bp flanking region. For each admixed individual, locus-specific ancestry for each gene was calculated as the proportion of *P. trichocarpa* ancestry, while chloroplast ancestry was assigned based on phylogenetic inference.

Geographic clines were modelled for chloroplast ancestry, 282 N-cp and 2611 non-interacting nuclear genes using the R package HZAR [53]. Cline parameters, including center and width (calculated as 1/maximum slope), were used to compare introgression across groups. For each contact zone, geographic distance from the sampling location closest to the coast for each genotype was calculated using the Haversine formula and normalized between 0 and 1 for comparison (electronic supplementary material, Methods). Clines were modelled and compared for different scale and tail parameters with Akaike’s Information Criterion (AIC) used to select the best fit model (electronic supplementary material, Methods). Cline parameters were compared between N-cp genes, chloroplast genome and non-interacting nuclear genes to test expectations of co-introgression. Co-introgression was observed when N-cp gene cline parameters overlapped with the 95% confidence interval (CI) of the chloroplast cline parameters, but not with the 95% CI for non-interacting nuclear genes. To determine whether the pattern of introgression for N-cp genes differs significantly from either chloroplast or non-interacting nuclear genes, we fit two constrained models for each N-cp gene in which the cline center was fixed within the 95% CI of either the chloroplast or non-interacting nuclear genes cline center using HZAR [53]. We then performed likelihood ratio tests (LRTs; 1 df) to assess whether the best-fit model for each N-cp gene differed significantly from either constrained model.

### (e) Common garden experiments

**Figure 1.**
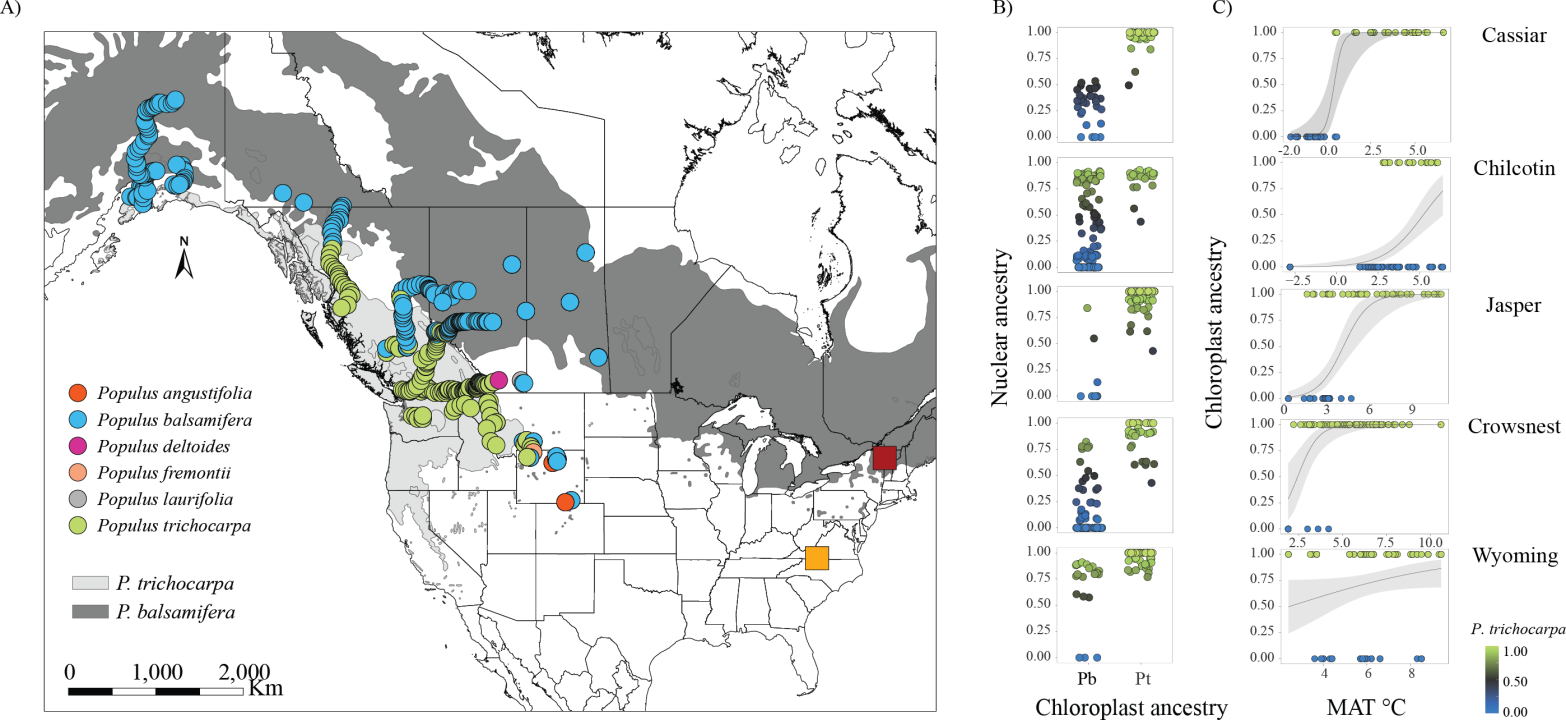
(A) Genotypes were collected across six east–west contact zones within the natural hybrid zone between *P. trichocarpa* and *P. balsamifera*. Light and dark grey represent the distribution range of *P. trichocarpa* and *P. balsamifera*, respectively. Georeferenced sampling locations are colour coded by chloroplast ancestry, *P. trichocarpa* (green), *P. balsamifera* (blue), *P. angustifolia* (orange), *P. deltoides* (purple), *P. fremontii* (peach), and *P. laurifolia* (grey). Common gardens were planted at Virginia Tech (yellow square) and University of Vermont (red square). (B) Chlorotype distribution (Pb: *P. balsamifera* or Pt: *P. trichocarpa*) associated with nuclear ancestry per contact zone. Alaska was excluded due to chloroplast capture. Each point represents a genotype, coloured by nuclear ancestry from *P. balsamifera* (blue) to *P. trichocarpa* (green) with admixed ancestry in grey. (C) Relationship between mean annual temperature (MAT) with chloroplast ancestry (0 = *P. balsamifera*, 1 = *P. trichocarpa*). Points are colour-coded by chloroplast ancestry. The grey line represents the predicted probabilities of chloroplast ancestry based on the fitted logistic regression with the shaded areas indicating 95% confidence intervals.

A total of 574 *Populus* genotypes were clonally propagated and planted in March 2020 in two common gardens located in Stuart, VA (36°37′ N, 80°09′ W; 359 m) and Burlington, VT (44°26′ N, 73°11′ W; 130 m; figure 1). Each garden was established using a randomized complete block design with three replicates per genotype, totaling 1722 trees per garden. During summer 2022, we measured stomatal conductance (*g*_sw_, mmol m⁻² s⁻¹), chloroplast fluorescence parameters including quantum efficiency of photosystem II (ΦPSII), minimum fluorescence (Fs), maximum fluorescence (Fm) and electron transport rate (ETR, µmol m⁻² s⁻¹), as well as leaf carbon isotopes (δ¹³C, ‰) and nitrogen content (N, %; details in electronic supplementary material, Methods).

### (f) Heritability

To quantify the proportion of phenotypic variance explained by genetic differences among genotypes, we estimated broad-sense heritability (*H*²) for each trait across each common garden. A linear mixed-effects model for each garden was fitted using equation (2.1):


(2.1) $$\;y_{ijk}=\mu +G_{i}+E_{j}+\;\varepsilon_{ijk},$$

where $y_{ijk}$ is the observed trait value for the individual *k* in the genotype *i* and block *j*. *G_i_* is the random effect of genotype representing the genetic effects (*V_G_*), *E_j_* is the random effect of block representing environmental effects (*V_E_*) and *ε_ijk_* is the residual error (*V_ε_*). Variance components from the model were extracted using VarCorr in R v. 4.3.1. *H*² was then calculated using equation (2.2):


(2.2) $$H^{2}=\;\frac{V_{G}}{V_{G}+V_{E}+V_{\varepsilon }}.$$

### (g) Statistical analysis

To quantify the effect of the interaction between nuclear and chloroplast ancestry to physiological trait variation across environments, each trait was modelled using equation (2.3):


(2.3) $$y_{ijkl}=\mu +N_{i}+C_{j}+N_{i}\;\times \;C_{j}+E_{k}+R_{l\left(k\right)}+\;\varepsilon_{ijkl}.$$

where $N_{i}$ is the fixed effect of nuclear genome ancestry *i*, $C_{j}$ is the fixed effect of chloroplast ancestry *j* and $N_{i}\;\times \;C_{j}$ corresponds to the interaction between them. $E_{k}$ is the fixed effect of common garden environment *k* accounting for environmental differences across common garden experiments. The random effect of block nested within garden $R_{l(k)}$ accounts for environmental variation within garden and *ε_ijkl_* is the error term. A second model was tested to include the interaction between chloroplast ancestry and environment, as well as the three-way interaction among environment, nuclear, and chloroplast ancestry. However, these interactions were not significant, so the simplified model was used (electronic supplementary material, Methods). Models were fitted using the package lme4 [54] in R v. 4.3.1. For each model, normality of residual errors was visually assessed.

## 3. Results

### (a) Chloroplast ancestry and geographic distribution across the hybrid zone

Phylogenetic analysis of 563 genotypes indicated that 247 genotypes had a chlorotype associated with *P. trichocarpa* and 298 genotypes had the *P. balsamifera* chlorotype (electronic supplementary material, figure S2). Of the remaining 18 genotypes, a BLASTn comparison of *Populus* chloroplast genomes revealed varying ancestry with 99% similarity with either *P. laurifolia* (5), *P. deltoides* (1)*, P. fremontii* (4) or *P. angustifolia* (8; electronic supplementary material, figure S3). In addition, across contact zones between *P. trichocarpa* and *P. balsamifera*, the distribution of chlorotypes varied. In the northern Alaska contact zone, *P. balsamifera* was the sole chlorotype observed, indicating a likely chloroplast capture (figure 1). Both chlorotypes were observed across all other contact zones; however, the *P. balsamifera* chlorotype extended across the entire geographic extent of the Chilcotin contact zone.

### (b) Chloroplast genome structure and genetic variation

Chloroplast structure was highly conserved for both chlorotypes, showing the typical quadripartite organization with two inverted repeats separated by large and small single-copy regions and 78 coding genes (electronic supplementary material, figure S4). A comparison of the *P. trichocarpa* and *P. balsamifera* chlorotypes identified 62 chlorotype-specific differences with 9 variants resulting in an amino acid change (electronic supplementary material, table S4). These nonsynonymous substitutions occurred within the following chloroplast genes: *matK* (1), *ndh*D (1), *ndh*K (1), *rpo*A (1), *rpo*C2 (1), *rps*8 (1) and *ycf*1 (3). These results suggest that while gene content is conserved between chlorotypes, there are chlorotype-specific differences that may underlie functional variation. To assess the ancestral state of the fixed amino acid substitutions, we reconstructed gene trees based on codon alignments. In general, the *P. trichocarpa* chlorotype aligned with the outgroup species, suggesting a shared ancestral lineage for the codon, with the exception of the gene *ndh*K. In contrast, the *P. balsamifera* chlorotype consistently formed a separate derived clade (electronic supplementary material, table S5).

**Figure 2.**
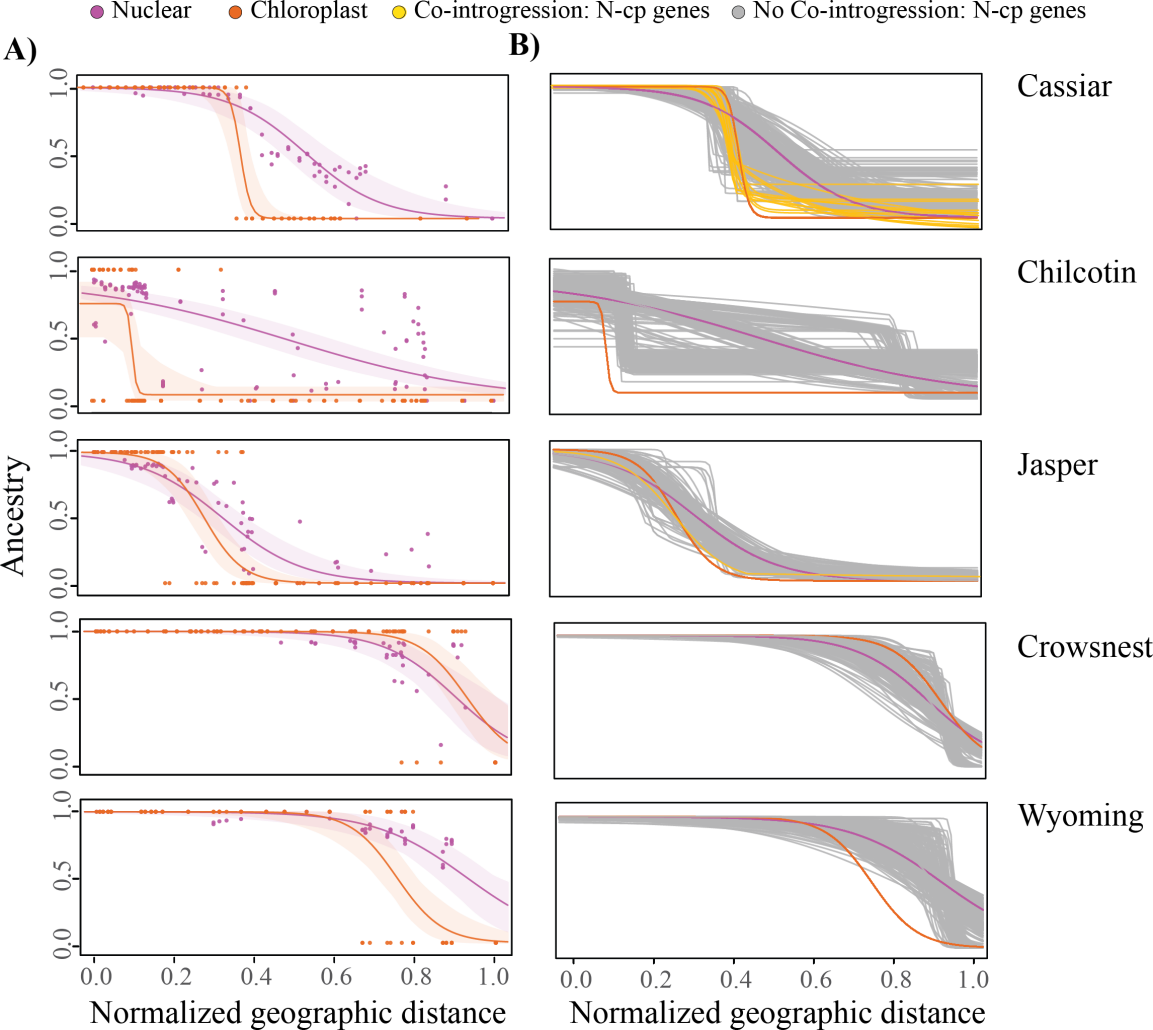
(A) Maximum likelihood geographic clines for chloroplast ancestry (orange line) and mean non-interacting nuclear genes (purple line) across five contact zones. Alaska was excluded due to chloroplast capture. The *x*-axis represents scaled distance from the coast and y-axis represents local ancestry (0 = *P. balsamifera* or 1 = *P. trichocarpa*). Modelled relationships and 95% confidence intervals for the best fit model are shown, with points representing individual genotypes. (B) Co-introgression tests for nuclear–chloroplast (*N-cp*) genes and chloroplast across contact zones. The best fit geographic cline model for each N-cp genes (yellow = co-introgression and grey = no co-introgression), chloroplast ancestry (orange) and mean non-interacting nuclear genes (purple) is shown.

We compared chloroplast and nuclear ancestry across contact zones. Notably, while individuals with the *P. balsamifera* chlorotype were observed across the full extent of nuclear genome ancestry, the *P. trichocarpa* chlorotype was restricted to individuals with 42% to 100% *P*. *trichocarpa* nuclear ancestry across all six contact zones (figure 2A). This suggests that barriers to *P. trichocarpa* chloroplast introgression may exist where the nuclear genome for *P. balsamifera* is most prevalent.

### (c) Differential influence of climate on chloroplast ancestry across repeated contact zones

Logistic regression models revealed that chloroplast ancestry was strongly associated with climatic gradients, although the strength and direction of these associations varied across contact zones (figure 1C and electronic supplementary material, figure S5). Overall, MAT, TD and RH were the strongest predictors for chlorotype across the contact zones with *P. trichocarpa* chlorotype more common in warmer, wetter environments and less continental environments relative to the *P. balsamifera* chlorotype (electronic supplementary material, table S6). In the Cassiar contact zone, sharp transitions associated with MAT (*slope* = 4.23 per °C, *p* < 0.001) and RH (*slope* = 1.18 per %, *p* < 0.001) were observed with *P. balsamifera* chlorotypes restricted to colder (−0.2 to 0.6°C MAT) and drier (56−63% RH) environments, while *P. trichocarpa* chlorotypes occupied warmer (0.6−6.6°C MAT) and increasingly humid (62−75% RH) regions (electronic supplementary material, figure S5). The probability of *P. trichocarpa* presence decreased with TD (*slope* = −1.17 per °C, *p* < 0.001) and CMD (*slope* = −0.02 per mm, *p* < 0.001) but increased with MAP (*slope* = 0.02 per mm, *p* < 0.001) and PAS (*slope* = 0.01 per mm, *p* < 0.001, electronic supplementary material, figure S5), suggesting that climate is likely a selective force shaping the chloroplast distribution across the Cassiar contact zone.

Similar associations were observed in the Chilcotin, Jasper and Crowsnest contact zones, where *P. trichocarpa* chloroplast presence increased with warmer (*slope* = 0.85−1.69 per °C, *p* < 0.001) and less continental (*slope* = −0.48 to −1.12 per °C, *p* < 0.001) environments, although the strength of these associations was lower with respect to Cassiar (electronic supplementary material, table S6). Additionally, RH was positively associated with *P. trichocarpa* presence in Crowsnest and Jasper (*slope* = 0.37−0.41 per %, *p* < 0.001) but had a negative effect in Chilcotin (−0.19 per %, *p* < 0.01). Importantly, the *P. balsamifera* chlorotype had a narrower climatic distribution in Jasper and Crowsnest.

In the Wyoming contact zone, temperature and continentality did not influence chloroplast ancestry distribution but the *P. trichocarpa* chloroplast increased with RH (*slope =* 0.21 per %, *p* < 0.001), MAP (*slope* −0.01 per mm, *p* < 0.001) and PAS (*slope* = 0.01 per mm, *p* < 0.01) pointing towards the influence of moisture rather than temperature as selective force within this contact zone. Generally, *P. balsamifera* chlorotype tended to occupy a narrower range of RH, MAP and PAS values within this contact zone. These results indicate that chloroplast ancestry is influenced by climate but the strength and direction of these associations vary by contact zone.

### (d) Directional introgression of the *P. balsamifera* chlorotype with limited evidence of co-introgression between chloroplast- and nuclear-interacting genes

A comparison of geographic clines revealed directional chloroplast introgression, with *P. balsamifera* chloroplasts moving into the *P. trichocarpa* geographic range (figure 2). In Alaska, there was a chloroplast capture for the *P. balsamifera* chlorotype; therefore, no clinal gradients were assessed. In the Cassiar contact zone, a steep, localized transition between chlorotypes was observed (*w* = 0.05, *c* = 0.43; figure 2A; electronic supplementary material, table S7), suggesting potential barriers to chloroplast introgression. Interestingly, 12 of 234 N-cp genes co-introgressed with the chloroplast within this contact zone (figure 2B). These included genes involved in RNA processing, proteolysis, photosystem I, ribosomal proteins and transfer RNAs (electronic supplementary material, table S8), suggesting that where co-introgression was observed it included those N-cp genes involved in essential chloroplast function.

Across the Chilcotin and Jasper contact zones, chloroplast clines were wider (*w* = 0.04, 0.19; figure 2A) consistent with weak barriers to gene flow. Cline centers were geographically located within the range of distribution of *P. trichocarpa* (*c* = 0.10, 0.27) suggesting introgression of the *P. balsamifera* chlorotype. In general, N-cp genes did not exhibit concordant cline centers or widths with the chloroplast genome, except for PNSB3 in Jasper, suggesting a lack of co-introgression between chloroplast and N-cp genes (figure 2B). Both the Crowsnest and Wyoming contact zones exhibited the widest chloroplast clines (*w* = 0.24, 0.23; *c* = 0.93, 0.75), with the transition between chlorotypes occurring towards the geographic interior of the hybrid zone (*c* = 0.93, 0.75). There was no evidence of co-introgression between the chloroplast and N-cp genes suggesting that introgression of N-cp genes is independent of the chloroplast within these contact zones.

### (e) Cytonuclear interactions influence physiological traits

Traits related to chloroplast function (ΦPSII, Fm, Fs and ETR) exhibited low heritability values (0.00–0.08; electronic supplementary material, table S9) regardless of the planting environment. For the Vermont and Virginia common garden, water-use efficiency (δ^13^C, 0.21–0.28), nitrogen content (0.13–0.22) and *g*_sw_ (0.17–0.32) had higher heritability estimates indicating increased genetic contribution to variation in these traits independent of the planting environment.

**Figure 3.**
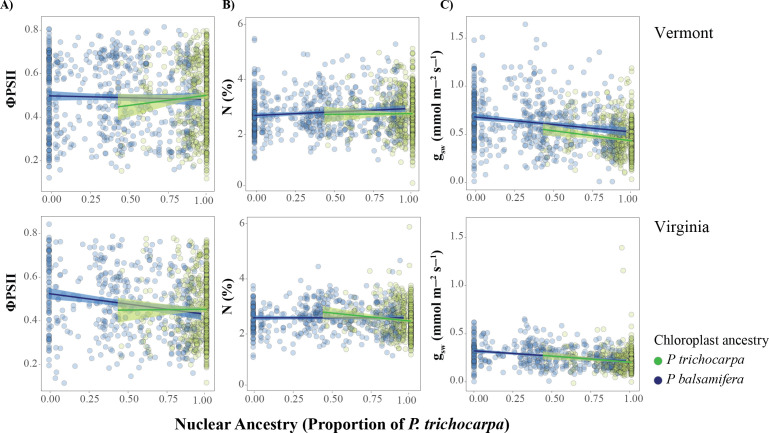
Relationships between (A) the quantum efficiency in photosystem II (ΦPSII), (B) nitrogen content (N), (C) stomatal conductance (*g*_sw_), and the interaction between nuclear and chloroplast ancestry across two environments. Points represent a *Populus* clone assessed within each environment. Lines represent regression model fit with 95% confidence intervals. Colours denote chloroplast ancestry, with blue for *P. balsamifera* and green for *P. trichocarpa*.

Cytonuclear interactions significantly affected efficiency of ΦPSII and Fm with mismatched nuclear and chloroplast ancestries reducing the efficiency of light absorbance (*p* < 0.01, *slope* = −0.11 and −52.65, respectively; figure 3A). Individuals with a *P. balsamifera* chlorotype had a slight efficiency advantage where nuclear ancestry was intermediate (50% *P. trichocarpa* ancestry). However, as *P. trichocarpa* nuclear ancestry increased beyond approximately 75%, individuals' genotypes with a mismatched chloroplast ancestry (*P. balsamifera* chloroplast) exhibited reduced efficiency. Interestingly, the negative effect of the interaction between nuclear and chloroplast ancestry on ΦPSII and Fm was similar across the two common gardens (*p >* 0.05, figure 3A). We also measured Fs, ETR and δ¹³C and noted no effect of either nuclear ancestry, chloroplast ancestry or their interactions on phenotypic variation (; electronic supplementary material, table S9 and figure S6). Overall, these results suggest that mismatched nuclear–chloroplast combinations can reduce performance for key photosynthetic traits across environments.

A significant positive effect of cytonuclear interactions on nitrogen content in leaves was detected with the interaction between nuclear and chloroplast ancestry (slope = 0.43, *p* < 0.05) indicating that concordant ancestry can increase nitrogen content (electronic supplementary material, table S9; figure 3B). If genotypes had 50% *P. trichocarpa* nuclear ancestry and the *P. trichocarpa* chlorotype, they exhibited increased nitrogen content relative to those that had the *P. balsamifera* chlorotype. Overall, nitrogen content in leaves was significantly reduced in the Virginia garden relative to the Vermont garden, with an average decrease of 0.24% (*p* < 0.001) indicating site-specific effects. These results suggest that nitrogen content can be impacted by mismatch in cytonuclear ancestry and local site-specific conditions.

While cytonuclear interactions did not influence *g*_sw_, a significant negative effect of both nuclear ancestry and common garden environment was observed (figure 3C). *g*_sw_ decreased at a rate of 0.16 mmol m⁻^2^ s⁻^1^ as the nuclear ancestry shifted from *P. balsamifera* towards *P. trichocarpa* (*p* < 0.001; electronic supplementary material, table S9 ) reflecting species-specific differences in water-use strategies. Furthermore, *g*_sw_ was significantly reduced in Virginia relative to Vermont, with an average decrease of 0.26 mmol m⁻² s⁻¹ in the Virginia garden (*p* < 0.001) indicating strong site-specific environmental effects. Chloroplast ancestry did not appear to have a significant effect on *g*_sw_ (figure 3C). These results indicate that *g*_sw_ is influenced by nuclear ancestry and environmental conditions rather than cytonuclear interactions.

## 4. Discussion

The theory of co-adaptation suggests the nuclear and cytoplasmic genomes have co-evolved to optimize physiological processes critical to plant fitness across environments [2,9,10,24,55]. However, hybridization can disrupt co-adapted gene complexes with a range of fitness consequences [22]. Across six contact zones between two sister *Populus* species, we found that nuclear–chloroplast (N-cp) genes often introgress independently of chloroplast ancestry. However, the extent of co-introgression varied by contact zone suggesting that local environmental conditions may play a role influencing patterns of co-introgression. Furthermore, common garden experiments revealed that chloroplast and nuclear mismatch can modify photosynthetic efficiency, although the effect of the chloroplast–nuclear mismatch can be environmentally dependent. Overall, our results suggest that chloroplast and nuclear genomes interact to influence adaptive trait variation. Given their importance for plant function, understanding how these interactions respond to climate change will be critical for predicting plant performance under future climates.

### (a) Chloroplast divergence and directional introgression are shaped by selection and demography

A comparison of chlorotypes for *P. trichocarpa* and *P. balsamifera* indicated chlorotype-specific differences associated with fixed amino acid changes*.* Despite only approximately 2% sequence divergence, nine fixed amino acid changes were identified in genes associated with water-use efficiency (*rpo*C2, *ycf*1 [56]), high-light oxidative stress (*ndh*D, *ndh*K [6,57]), cold tolerance (*rps*8 [58]) and splicing and biogenesis (*mat*K, *rpo*A). The maintenance of these chlorotype-specific differences suggests that they may underlie functional variation important to adaptive differentiation between the two species.

The distribution of chlorotypes across nuclear ancestry was highly asymmetric, suggesting differences in cytonuclear compatibility between species. The *P. trichocarpa* chloroplast was observed only in genotypes with 42–100% of *P. trichocarpa* nuclear ancestry, whereas the *P. balsamifera* chloroplast was observed across the full spectrum of nuclear ancestry. Similar cases of asymmetric cytonuclear incompatibility have been observed in other systems, where one cytoplasmic genome shows pronounced incompatibility with divergent nuclear backgrounds [23,59]. Phylogenetic inference indicated that *P. balsamifera* carries the derived allelic state across the majority of fixed chloroplast differences. If N-cp genes in *P. balsamifera* coevolved with the derived chloroplast genome, this chloroplast may maintain broad compatibility across nuclear backgrounds. Conversely, if the *P. trichocarpa* chloroplast reflects the ancestral state, its N-cp genes are likely to have evolved compatibility with the ancestral background, rather than with the more recently derived *P. balsamifera* genome. Such differences in compatibility could influence both the direction and magnitude of chloroplast introgression, potentially shaping the regional patterns of *P. balsamifera* chloroplast distribution observed across the six contact zones.

The geographic distribution of chlorotypes largely followed species-specific geographic distributions. However, we detected a chloroplast capture for *P. balsamifera* in the Alaska contact zone and evidence of *P. balsamifera* expansion into the range of *P. trichocarpa* in the Chilcotin contact zone. While selection may favour chlorotypes due to adaptation or cytonuclear compatibilities, neutral processes including direction of gene flow or demographic history, can also impact the distribution of chloroplast genomes [19,25,27,60]. The complete absence of pure *P. trichocarpa* chlorotypes in the Alaska contact zone suggests that *P. balsamifera* populations probably established first in this region. This is reflected in the persistence of *P. balsamifera* through the Last Glacial Maximum within northern Alaska and adjacent unglaciated regions of Beringia [38,39]. Following glacial retreat, northward expansion of *P. trichocarpa* populations along the Pacific Northwest coast likely contributed pollen needed to facilitate hybrid formation in Alaska. However, no pure *P. trichocarpa* individuals were observed in this transect. In Chilcotin, demographic processes, including seed-mediated gene flow, facilitated by landscape features such as river corridors [36] may be driving the expansion of the *P. balsamifera* chlorotype into historically *P. trichocarpa*-occupied areas. The strong association of the *P. balsamifera* chlorotype with colder and drier climatic conditions relative to *P. trichocarpa* suggests that this chlorotype may provide an adaptive advantage in colder environments. Although our models tested associations between chloroplast ancestry and climate alone, chloroplast ancestry often covaries with nuclear ancestry, and geography frequently covaries with climate due to postglacial range shifts in forest tree species [61]. Additionally, limited sampling within the *P. balsamifera* range may further influence these climate associations, potentially biasing the strength or direction of detected associations. While this limits our ability to fully tease apart climate-driven selection from demographic history, consistent associations with climatic variables suggest environment may act as an important selective force maintaining regional differences [62]. Thus, the contemporary distribution of chloroplast genomes probably results from interactions between historical demography, ongoing gene flow and environmental selection.

### (b) Co-introgression of chloroplast and nuclear–chloroplast genes results from intrinsic and extrinsic selection

Despite predictions that co-adapted genes should co-introgress following hybridization [31,63], our results show limited evidence of co-introgression. Co-introgression with the chloroplast genome was observed within the Cassiar contact zone for a subset of N-cp genes. The steep chloroplast cline in Cassiar suggests cytonuclear combinations are likely constrained by selection within this region, potentially due to both intrinsic incompatibilities and extrinsic selection. Co-introgressing genes in Cassiar are primarily associated with RNA processing, proteolysis, photosystem I, ribosomal proteins and transfer RNAs suggesting that cytonuclear coordination may be required for these essential chloroplast functions. Notably, four co-introgressing genes encode subunits of the *Clp*P protease complex, a relatively small plastid-localized enzyme involved in protein turnover [64]. This enrichment may reflect the need to preserve functional coordination within the complex. Although no fixed differences were found in the chloroplast *clp*P1 gene itself, one fixed variant upstream was detected that may play a regulatory role supporting localized cytonuclear coevolution (electronic supplementary material, table S4). Consistent with findings in other systems, co-introgression between cytoplasmic and nuclear genomes appears to be restricted to a limited number of genes, typically those critical for organelle performance, rather than occurring broadly across the genome [30,63].

In the Cassiar contact zone, the restricted pattern of co-introgression may reflect climate-driven selection, aligning with steep climate turnover from maritime to boreal conditions across this contact zone [65–68]. Chorotype distribution in this region was strongly associated with climatic variables, including MAT and RH, suggesting that environmental gradients may impact introgression. Such patterns are expected when nuclear and organellar genomes are adapted to local environments [31] as environmental gradients can reinforce barriers to gene flow by favouring cytonuclear combinations that optimize organelle functions [16]. Therefore, the limited co-introgression of N-cp genes observed in Cassiar may be shaped by environmental selection acting to maintain cytonuclear compatibility needed for adaptation to local environmental conditions. This contrasts with other contact zones where we did not observe a strong role of climate in shaping the distribution of the chlorotype.

Demographic processes may also play a role influencing cytonuclear introgression, particularly in regions where environmental selection is weak [69]. In the Chilcotin, Jasper, Crowsnest and Wyoming contact zones, we observed no evidence of co-introgression between chloroplast and N-cp genes and only weak associations between chloroplast ancestry and climate. In these regions, recombination has likely played a major role in breaking down co-inherited ancestry blocks over time, particularly in older contact zones with a repeated history of hybridization [69,70]. This process can decouple chloroplast genomes from nuclear genes that interact with them, weakening any signal of co-introgression even if such associations were present initially. Moreover, repeated cycles of range expansion and contraction driven by climatic oscillations [36] can lead to founder effects and genetic drift. These demographic processes can alter the frequency of specific chloroplast haplotypes or nuclear ancestry blocks by chance, either preserving or eliminating cytonuclear combinations independent of their fitness effects. In such contexts, mismatched cytonuclear combinations may persist not because they confer an adaptive advantage, but because selection against them is weak or absent. Consequently, cytonuclear mismatches may be neutral and maintained within hybrid populations. The fitness consequences of such mismatches may only become apparent under specific environmental stressors, such as drought, nutrient limitation or temperature extremes [71,72]. Thus, the lack of co-introgression observed in these zones likely reflects the combined influence of neutral demographic processes, particularly recombination, and historical range dynamics and environment-dependent selection.

While our study provides valuable insights into cytonuclear introgression, this analysis relies on the identification of recognized N-cp genes annotated in the *Arabidopsis* genome [50]. The Cytonuclear Molecular Interactions Reference database for Arabidopsis (CyMIRA) provides the most comprehensive list of cytonuclear interactions. However, it is possible that not all potential N-cp genes in *Populus* were captured in this study. Future research should focus on identifying *Populus*-specific N-cp genes by leveraging admixture mapping to detect regions of the genome associated with chloroplast ancestry. This could be followed by functional validation of candidate genes through expression analyses or transgenic approaches [73]. Furthermore, our study focuses on chloroplast–nuclear interactions but mitochondrial genomes also interact with the nuclear genome to regulate key cellular processes including respiration and stress responses [4,7,73,74]. Given that mitochondrial genes are also uniparentally inherited and subject to cytonuclear co-adaptation, future work should explore whether nuclear–mitochondrial interactions exhibit similar patterns of introgression.

### (c) Discordance between nuclear and chloroplast genomes reduce light absorbance

The nuclear and chloroplast genomes interact to influence traits important to adaptation including physiological traits critical to plant fitness [1,8,14,15]. The efficiency of photosystem II (ΦPSII) and maximum fluorescence (Fm) are particularly important during early tree establishment as reduced light absorbance limits photosynthetic capacity essential for survival and performance [75]. Here, we provide evidence that cytonuclear interactions negatively affect ΦPSII and Fm, with mismatched chloroplast and nuclear ancestry reducing their efficiency in two different environments. Generally, individuals with matched chloroplast and nuclear ancestry had higher light absorbance than those with mismatched ancestry. The higher light absorbance for individuals with matched genome ancestry highlights the importance of positive intergenomic epistasis for plant adaptation. For admixed genotypes, those carrying the *P. balsamifera* chlorotype had a slight advantage when nuclear ancestry was evenly mixed (approx. 50% *P. trichocarpa* or *P. balsamifera*). However, this advantage diminished as *P. trichocarpa* nuclear ancestry increased, suggesting that cytonuclear mismatches reduce the efficiency of light absorbance, particularly in hybrids with a *P. balsamifera* chlorotype and greater proportion of *P. trichocarpa* nuclear genome ancestry. These findings align with previous work in *Arabidopsis* [2], *Ipomopsis* [19], *Helianthus* [15] and wild barley [76] that showed mismatches between nuclear and chloroplast genome ancestry reduced the efficiency of key physiological traits. As the reduction in ΦPSII and Fm was observed in both common gardens, the effect is likely driven by intrinsic incompatibilities rather than an environment-specific stress response [31,71]. These incompatibilities may reflect Bateson–Dobzhansky–Muller (BDM) incompatibilities, which predict negative fitness outcomes associated with epistatic interactions between divergent alleles [21,22]. Our findings support the idea that *P. trichocarpa* and *P. balsamifera*, which diverged over approximately 2.12 million years ago [36] have evolved lineage-specific cytonuclear interactions, with phylogenetic evidence indicating that *P. balsamifera* carries the derived allelic state across the majority of fixed chloroplast differences. Disruptions of co-adapted interactions via hybridization may lead to the evolution of postzygotic barriers, limiting the direction and extent of introgression in hybrid zones.

In contrast to the consistent effects observed for light absorbance, the impact of cytonuclear interactions on nitrogen content in leaves was environment-specific. Nitrogen plays an essential role in the photosynthetic processes, contributing to the synthesis of light-harvesting proteins, bioenergetic molecules and enzymes like Rubisco that are critical for carbon fixation [77]. Matched nuclear and chloroplast ancestry were associated with increased leaf nitrogen content suggesting that cytonuclear concordance enhances nitrogen-related physiological function beyond its role in light absorbance. The positive effect of matching nuclear and chloroplast ancestry on nitrogen content was more pronounced in the Virginia garden, where overall nitrogen availability was significantly lower. This environmental-dependent response parallels patterns observed in *Ipomopsis* and *Penstemon* hybrids, where reciprocal hybrid performance varied across environments [71,72]. In both systems, the strength and direction of cytoplasmic effects shifted with habitat indicating that environmental selection likely modulates cytonuclear interactions. Differences in leaf nitrogen content across genotypes may reflect either variation in soil nitrogen availability or physiological efficiency in uptake and assimilation [19]. Virginia and Vermont common gardens differ in soil-use history; the Vermont site was formerly a maize field and likely has higher residual nitrogen. Reduced nitrogen content in mismatched genotypes in Virginia suggests that cytonuclear incompatibilities constrain nitrogen assimilation under environments with different soil conditions. These results suggest that cytonuclear interactions may shape hybrid zone dynamics through environmental selection against mismatched genotypes, thereby influencing patterns of introgression.

Broad-sense heritability (*H*²) captures the proportion of phenotypic variance explained by genetic factors including additive, dominance and epistatic effects. *H*² was low for ΦPSII and Fm for the common gardens despite clear evidence for cytonuclear effects on these traits. This suggests that most of the phenotypic variation in light absorbance traits is attributable to environmental or residual sources such as microclimatic variation within blocks, measurement error, unmodelled genotype-by-environment interactions or epigenetic effects, rather than genetic differences among genotypes. The low *H*² values for light absorbance traits may reflect plasticity to environmental heterogeneity within the gardens. This is further supported by the fact that *R*² marginal values, representing variance explained by fixed effects (ancestry and environment), were much lower than *R*² conditional values, which include both fixed and random effects (electronic supplementary material, table S9). This suggests that a substantial portion of trait variation is captured by block effects, and that unmeasured environmental factors or fine-scale variation may play a dominant role in shaping observed trait variation. In contrast, traits such as leaf nitrogen content, which showed a positive and significant cytonuclear interaction exhibited moderate heritability, suggesting that the beneficial effect of cytonuclear concordance on nitrogen content is at least partially heritable across environments. Other traits such as stomatal conductance (*g*_sw_) and water use efficiency (δ¹³C) also showed moderate heritability but did not exhibit significant cytonuclear interactions. This indicates that while these traits are influenced by genetics, variation observed is not mainly driven by cytonuclear interactions.

## 5. Conclusion

We evaluated the role of cytonuclear interactions to plant adaptation across environments. Chlorotype-specific differences may contribute to functional variation critical for adaptation. While we found some evidence of co-introgression between the chloroplast and N-cp genes, differential patterns of co-introgression across contact zones are likely shaped by demographic or environmental differences among contact zones. However, the degree of match or mismatch between chloroplast and nuclear genome ancestry can influence plant function across environments, with implications for adaptive evolution where sister species hybridize particularly under climate change. Because cytonuclear interactions may constrain physiological performance, studies assessing the adaptive capacity of species and their hybrids should account for these interactions when evaluating responses to environmental change.

## Data Availability

Genomic data used in this study from a previously published study by Bolte *et al.* [36] are archived on NCBI (PRJNA996882). NCBI accession data for chloroplast genomes are available in the supplementary material. All the scripts to replicate the analyses were deposited in Dryad [78]. Supplementary material is available online [79].
